# Shifts between pro-inflammatory and anti-inflammatory profiles in pregnant mares: a review of physiological functions

**DOI:** 10.3389/fvets.2025.1660759

**Published:** 2025-09-18

**Authors:** Katiuska Satué, Deborah La Fauci, Pietro Medica, Elena Damiá Gímenez, Cristina Cravana, Esterina Fazio

**Affiliations:** ^1^Department of Animal Medicine and Surgery, Faculty of Veterinary Medicine, CEU-Cardenal Herrera University, Moncada, Spain; ^2^Department of Veterinary Sciences, Polo Universitario Annunziata, Messina, Italy

**Keywords:** mare, pregnancy, immune regulation, conceptus mobility, regulatory T cells, maternal-fetal tolerance

## Abstract

Pregnancy in the mare presents a unique immunological challenge, requiring a finely tuned balance between pro-inflammatory and anti-inflammatory responses to ensure reproductive success. Throughout gestation, the maternal immune system undergoes dynamic adaptations to support key reproductive events from conceptus mobility and fixation to the formation and function of endometrial cups, and the expansion of immunoregulatory cells that promote maternal-fetal tolerance. In early pregnancy, a controlled pro-inflammatory environment facilitates critical processes such as embryo migration and implantation. As gestation progresses, the immune profile shifts toward a tolerogenic state, marked by the proliferation of regulatory T cells (Tregs) and the activity of tolerogenic antigen-presenting cells. These adaptations protect the semi-allogeneic conceptus and sustain pregnancy. Toward term, a resurgence of pro-inflammatory signaling becomes essential to initiate parturition, activating pathways that promote uterine contractility, cervical ripening, and fetal expulsion—demonstrating that inflammation is not only a threat but a physiological necessity. Disruptions in this immunological balance whether due to excessive inflammation or insufficient tolerance can compromise embryonic and fetal viability, increasing the risk of pregnancy loss. Therefore, a comprehensive understanding of the immunological and inflammatory mechanisms throughout equine gestation is essential to advance reproductive physiology and improve clinical strategies for fertility management in mares.

## 1 Introduction

Pregnancy is a physiological condition involving a series of tightly regulated processes that occur sequentially across distinct stages. These processes require coordinated endocrine, immunological, and structural adaptations within the uterus. The gestational sequence begins with fertilization of the ovum, followed by implantation of the blastocyst into the maternal endometrium. For implantation to occur successfully, the blastocyst must adhere to the endometrial surface to establish access to oxygen and nutrients essential for early embryonic development (1). These physiological changes involve extensive tissue remodeling and activation of inflammatory pathways within the uterus. In the mare, pregnancy constitutes an immunologically dynamic state that demands a finely regulated balance between pro-inflammatory and anti-inflammatory immune responses. This equilibrium is essential to support fetal development while maintaining maternal immune competence (2). Pregnancy in the mare is marked by dynamic and tightly regulated modifications in the maternal immune response throughout the various stages of gestation. The sequential shifts between pro-inflammatory and anti-inflammatory immune profiles are critical for facilitating embryo implantation, promoting placental development, establishing fetal immune tolerance, and ultimately enabling successful parturition.

Table 1 provides an overview of the key immunological phases of equine pregnancy, emphasizing the predominant immune responses and their associated physiological functions. A key factor shaping the early inflammatory environment is maternal exposure to seminal plasma, which plays a pivotal role in modulating immune responses to promote embryo acceptance and facilitate successful establishment of pregnancy.

**Table 1:** Summary of key immunological and molecular events during equine pregnancy, highlighting the phases of the inflammatory response and their associated mediators.

Pregnancy stage	Early pregnancy (0–40 days)	Mid-pregnancy (40–250 days)	Late pregnancy (250–340 days)
Key Processes	Implantation and Maternal Recognition	Fetal Tolerance and Placental Support	Parturition and Cervical Ripening
Cytokines	↑ IL-1β, TNF-α, IL-6	↑ IL-10, TGF-β	↑ IL-6, IL-8, PGF2α
Immune Cells	↑ Neutrophils, Macrophages, DCs, uNK cells	↑ Macrophages, DCs, uNK cells, Regulatory T cells (Tregs)	↑ Macrophages, Neutrophils, DCs, Tregs, Effector T cells
Other Mediators	↑ Prostaglandins (PGE2, PGF2α) ↑LIF, OPN, integrins	↑ IGFs, FGFs ↑ TIMPs	↑ Inflammatory mediators for labor (IL-1β, TNF-α, prostaglandins)
Immune Profile	Pro-inflammatory (Th1-dominant)	Anti-inflammatory (Th2/Treg-dominant)	Pro-inflammatory (Th1-dominant for labor)

Source: Data from Figarska and Witkowska-Piłaszewicz (2).

### 1.1 Role of stallion seminal plasma in modulating maternal inflammation in mares

The deposition of semen into the uterus initiates multiple maternal immune responses involving both innate and adaptive immunity (3, 4). Seminal plasma is a complex biological fluid that contains a wide spectrum of bioactive molecules with potential roles in modulating reproductive processes. Among these are cytokines such as Transforming Growth Factor beta (TGF-β), Interleukin-6 (IL-6), and Interleukin-8 (IL-8); hormones including prostaglandins; paternal antigens; and immunomodulatory factors such as colony-stimulating factor 2 (CSF2) and tumor necrosis factor-related apoptosis-inducing ligand (TRAIL). In addition, seminal plasma carries transcripts associated with embryonic development and metabolic regulation, notably insulin-like growth factor beta (IGF-β) and plasma-derived cysteine-rich secretory protein 3 (CRISP3) (5–7). The interaction between seminal plasma and the maternal reproductive tract initiates adaptive immune responses that promote tolerance to the semi-allogeneic fetoplacental unit, thereby supporting successful implantation and pregnancy maintenance (6, 8). Seminal plasma plays a pivotal role in modulating the uterine environment by inducing the production of cytokines and chemokines from epithelial cells, promoting the recruitment of innate immune cells—including neutrophils, macrophages, and dendritic cells (DCs)—and facilitating DC activation through the presentation of paternal antigens (9–11). By orchestrating inflammatory and regulatory signals, these innate immune cells contribute to the development of a uterine microenvironment that supports embryo implantation and promotes maternal immune tolerance toward the developing conceptus. Neutrophils, traditionally recognized for their antimicrobial activity, are among the earliest immune cells to respond to hormonal and embryonic cues within the uterine environment. Their defense mechanisms include phagocytosis and the release of neutrophil extracellular traps (NETs), which effectively neutralize invading pathogens. In addition to their protective functions, neutrophils secrete cytokines, chemokines, and proteolytic enzymes that initiate a tightly regulated pro-inflammatory response. This response is critical for endometrial remodeling during early pregnancy, while mechanisms are simultaneously engaged to minimize tissue damage and preserve uterine integrity (12). Through the secretion of cytokines, chemokines, and proteolytic enzymes, neutrophils initiate a finely regulated pro-inflammatory response that is essential for endometrial remodeling, while concurrently limiting tissue damage (13, 14). Moreover, neutrophil-derived mediators may contribute to extracellular matrix (ECM) turnover, thereby enhancing uterine receptivity and supporting the establishment of a favorable environment for embryo implantation (15).

Macrophages play a critical role in immune regulation at the maternal–fetal interface. By secreting growth factors and matrix metalloproteinases (MMPs), they contribute to angiogenesis and tissue remodeling, thereby supporting the structural and vascular adaptations of the endometrium required for successful implantation and pregnancy maintenance (16). Their immunomodulatory function is mediated through the production of anti-inflammatory cytokines, including IL-10 and TGF-β, as well as the expression of indoleamine 2,3-dioxygenase (IDO). These mechanisms collectively suppress T-cell activation and foster the expansion of regulatory T cells (Tregs), contributing to immune tolerance at the maternal–fetal interface (17). Collectively, these processes help maintain maternal tolerance to the semi-allogeneic conceptus.

Dendritic cells (DCs), the principal antigen-presenting cells within the uterine environment, undergo functional adaptations during early gestation. They present fetal antigens in a tolerogenic context and secrete IL-10, thereby promoting the differentiation of Tregs and contributing to maternal–fetal immune tolerance (17). DCs contribute to maternal–fetal immune tolerance by downregulating their ability to activate effector T cells, thereby preventing maternal immune rejection of the conceptus. Collectively, innate immune cells coordinate a finely tuned balance between immune activation and tolerance, ensuring maternal protection without compromising embryonic development. Seminal plasma components—particularly those originating from the seminal vesicles, such as CD38, play a pivotal role in modulating the maternal immune response, facilitating embryo recognition and the establishment of pregnancy. In other species, CD38 has been implicated in the induction of tolerogenic DCs and the expansion of CD4^+^FOXP3^+^ Treg cells, both of which are essential for sustaining immune tolerance at the maternal–fetal interface. In the mare, this immunological environment is notably complex and dynamic, characterized by a balance between Th1 and Th2 cytokine profiles and a regulatory T cell population that modulates immune interactions throughout gestation (18). A deficiency in Treg cells has been linked to implantation failure, inadequate uterine vascular remodeling, and increased risk of fetal loss during late gestation (18). Although equine-specific studies remain limited, emerging evidence indicates that stallion seminal plasma promotes the expansion of Treg cells, including the CD4^+^CD25^+^FOXP3^+^ subset, both systemically and within the endometrial tissue. These cells contribute to the establishment of a tolerogenic uterine environment by suppressing pro-inflammatory responses and facilitating maternal adaptation to the semi-allogeneic embryo. Furthermore, seminal plasma has been shown to modulate endometrial gene expression in mares, suggesting potential epigenetic influences on embryonic development and the phenotype of the offspring (19–21). Moreover, repeated exposure to seminal plasma from the same stallion may potentiate these immunomodulatory effects, potentially contributing to the observed variability in pregnancy outcomes across different mare–stallion pairings (11). Taken together, these observations highlight the pivotal role of stallion seminal plasma in shaping maternal immune responses that favor the establishment and maintenance of early pregnancy in mares.

Table 2 summarizes the principal immunomodulatory mechanisms of stallion seminal plasma during early pregnancy in the mare. These mechanisms involve a complex interplay of cytokines, growth factors, and immune cell modulation that promote maternal immune tolerance and create a uterine environment conducive to embryo implantation and development. Understanding these key factors is essential for appreciating how seminal plasma contributes to successful equine reproduction. Immune responses are fundamental throughout equine pregnancy, orchestrating key reproductive events such as embryo recognition, implantation, placentation, and parturition (22). As gestation advances, the maternal immune milieu gradually shifts toward an anti-inflammatory profile, characterized by elevated levels of cytokines such as L-10, TGF-β, and IL-4, along with the expansion of Treg cells. These immunological adaptations are crucial for establishing and maintaining fetal tolerance, primarily by suppressing cytotoxic T cell activity and modulating the function of uterine natural killer (uNK) cells (18). In the final stages of pregnancy, the immune system undergoes a transition back to a pro-inflammatory state. This resurgence, marked by increased concentrations of IL-6, IL-8, TNF-α, and prostaglandins (PGs), promotes cervical ripening, myometrial contractions, and fetal membrane rupture, all of which are essential for successful parturition (11).

**Table 2:** Key immunomodulatory mechanisms of seminal plasma during early pregnancy in the mare.

Mechanism	Description
Induction of Treg cells	Expansion of CD4^+^CD25^+^FOXP3^+^ Tregs in the endometrium and systemically, supporting maternal tolerance to the conceptus.
Activation of Tolerogenic DCs	Seminal plasma—especially soluble CD38—stimulates dendritic cells toward a tolerogenic phenotype, enhancing Treg induction.
Modulation of Cytokine and Chemokine Expression	Upregulation of anti-inflammatory cytokines (e.g., TGF-β, IL-10) and chemokines create a controlled inflammatory uterine environment.
Recruitment of Innate Immune Cells	Neutrophils, macrophages, and dendritic cells are recruited post-mating, aiding tissue remodeling and immune adaptation.
Stimulation of Embryokine Production	Increased expression of embryotrophic factors such as LIF, IL-6, CSF2, and TRAIL supports embryo development and implantation.
Modulation of Endometrial Gene Expression	Transcriptional changes in the endometrium shape embryo–uterine interactions and may influence offspring phenotype via epigenetic regulation.
Improved Embryo Development	Endometrial gene expression promotes immune tolerance and growth factor signaling, enhancing embryo viability and implantation success.

Source: Data from Guerin et al. (6); Doty et al. (7); Robertson et al. (8); Bromfield (19); Troedsson et al. (20); Fedorka et al. (26).

## 2 Immunological and molecular mechanisms of endometrial receptivity, embryo mobility, and implantation in the Mare

A distinctive aspect of equine reproductive physiology is the unusually prolonged pre-implantation period. Following ovulation, the equine conceptus enters the uterus between days 6 and 6.5 and continues to migrate throughout the uterine lumen until approximately day 16 (1). During early pregnancy in the mare, a transient pro-inflammatory response occurs shortly after insemination, associated with post-breeding clearance mechanisms triggered by exposure to seminal plasma and spermatozoa, as discussed in the preceding section. Between the post-insemination clearance phase and the onset of implantation, there is currently no conclusive evidence in the literature confirming or refuting the presence of a sustained pro-inflammatory state. In fact, an inflammatory uterine environment during embryonic migration would likely be detrimental, as it could induce luteolysis and lead to a return to estrus. Instead, conceptus mobility during this phase is thought to be supported by finely tuned endocrine and paracrine signaling particularly through the modulation of PG secretion. The active migration of the equine conceptus within the uterine lumen elicits localized inflammatory responses that help regulate PG secretion, maintaining a delicate balance between luteolytic prostaglandin F2α (PGF2α) and luteoprotective prostaglandin E2 (PGE2). This balance favors luteal maintenance and facilitates the establishment of pregnancy. The underlying regulatory mechanism is believed to involve an as-yet unidentified embryonic signal that inhibits the upregulation of oxytocin receptors and cyclooxygenase-2 (COX-2) expression, thereby limiting PGF2α synthesis. As a result, the corpus luteum (CL) remains functionally active and continues to secrete progesterone (P4), a hormone essential for inducing endometrial receptivity and sustaining early pregnancy (4, 22, 23). A second wave of immune activation arises closer to implantation, particularly after the emergence of the chorionic girdle (post day 20), and becomes more pronounced around day 40 of gestation, contributing to maternal recognition and placental development (22).

Beyond their luteal effects, prostaglandins such as PGE2 and PGF2α, along with other inflammatory mediators, orchestrate immune cell recruitment and induce critical endometrial modifications necessary for embryo implantation (2). These bioactive lipids, locally synthesized within the endometrium, upregulate adhesion molecules on uterine endothelial cells, facilitating the migration of immune cells—including macrophages, neutrophils, and DCs cells—to the implantation site. PGE2 promotes anti-inflammatory cytokine production and fosters immune tolerance, while PGF2α regulates vascular tone and stimulates myometrial contractions. Together, they activate MMPs to remodel the ECM, support trophoblast invasion, and stimulate local angiogenesis, enhancing blood flow and nutrient delivery to the developing conceptus. The coordinated actions of PGE2 and PGF2α thus establish an optimal immunological and structural environment, critical for embryo attachment and the maintenance of early pregnancy (24). During the early stages of equine pregnancy, particularly between days 9 and 16, a finely tuned inflammatory response within the endometrium plays a pivotal role in facilitating conceptus mobility, MRP, and preparation for embryo fixation. This immunological environment promotes the recruitment and activation of key immune cells including neutrophils, macrophages, and DCs mediated by a network of cytokines and chemokines. Interleukin-8 (IL-8/CXCL8) is instrumental in attracting and activating neutrophils, thereby contributing to tissue remodeling and local immune defense. Chemokine CCL2 (monocyte chemoattractant protein-1, MCP-1) supports the recruitment of monocytes and macrophages, which are essential for clearing cellular debris and facilitating ECM remodeling. CCL5 (RANTES) draws lymphocytes and other immune cells to the uterine environment, modulating the local immune response. Additionally, interferon-gamma (IFN-γ) enhances macrophage activation and regulates the expression of MHC molecules, helping to balance immune activation with tolerance at the maternal–fetal interface. These immune mediators work in concert to generate a responsive yet regulated inflammatory setting, essential for guiding conceptus movement, preserving luteal integrity, and preparing the uterine lining for embryo attachment (2, 18).

At the same time, pro-inflammatory cytokines such as IL-1β and TNF-α stimulate intracellular signaling cascades in endometrial cells, notably the Nuclear Factor kappa B (NF-κB) and Mitogen-Activated Protein Kinase (MAPK) pathways. This signaling activity induces the expression of adhesion molecules, particularly integrins transmembrane receptors that mediate interactions between cells and the ECM. These integrin-mediated connections are essential for securing the embryo to the uterine epithelium, facilitating stable attachment and progression toward implantation (1, 25, 26).

Table 3 provides an overview of key pro-inflammatory cytokines and chemokines involved in promoting successful early pregnancy in the mare.

**Table 3:** Key pro-inflammatory cytokines and chemokines involved early pregnancy in the mare.

Cytokine/Chemokine	Main Function(s)	Role during Early Pregnancy in Mare
IL-1β	Induces inflammation, activates immune cells	Stimulates leukocyte recruitment and endometrial remodeling
TNF-α	Promotes inflammation, apoptosis, and immune activation	Enhance prostaglandin synthesis and tissue remodeling
IL-6	Dual pro-inflammatory and anti-inflammatory mediator	Modulates immune response and supports trophoblast invasion
IL-8 (CXCL8)	Chemotactic factor for neutrophils	Recruits’ neutrophils to the endometrium
CCL2 (MCP-1, Monocyte Chemoattractant Protein-1)	Chemotactic for monocytes/macrophages	Attracts macrophages for tissue remodeling
CCL5 (RANTES)	Attract T cells, eosinophils, basophils	Supports immune cell recruitment to implantation site
IFN-γ	Activates macrophages, enhances antigen presentation	Modulates immune tolerance and supports immune surveillance

These molecules mediate immune cell recruitment, tissue remodeling, and modulation of endometrial receptivity, all of which are essential for embryo development and maternal recognition of pregnancy. IL = interleukin; TNF = tumor necrosis factor; CCL = chemokine (C-C motif) ligand; CXCL = chemokine (C-X-C motif) ligand; MCP = monocyte chemoattractant protein; RANTES = regulated on activation, normal T cell expressed and secreted. Source: Data from Allen et al. (1); Figarska and Witkowska-Piłaszewicz (2); Fedorka et al. (18); Fedorka et al. (26); Gibson et al. (25).

In addition, the inflammatory milieu promotes the secretion of implantation-associated proteins, such as osteopontin (OPN) and leukemia inhibitory factor (LIF). OPN functions as a ligand for integrins, reinforcing the adhesion between the trophoblast and the endometrial surface. LIF facilitates implantation by promoting trophoblast invasion and modulating immune tolerance, thereby contributing to a receptive uterine environment for the semi-allogeneic embryo. The coordinated upregulation of integrins, along with increased secretion of OPN and LIF, enhances endometrial receptivity by enabling stable embryo attachment and initiating controlled invasion into maternal tissue. This tightly regulated inflammatory process ensures successful implantation while preventing excessive immune activation that could compromise pregnancy (2). Crucially, the inflammatory response is precisely controlled to avoid excessive tissue damage and prevent immune rejection of the semi-allogeneic conceptus. As pregnancy advances, P4 serves as a central immunomodulatory hormone, downregulating the production of pro-inflammatory cytokines and fostering immune tolerance. Any disturbance in this finely tuned balance may compromise endometrial receptivity, potentially leading to implantation failure or early embryonic loss (EEL) (2).

### 2.1 Natural killer (NK) cells and uterine remodeling

Natural killer (NK) cells are key effectors of the innate immune system, known for their cytotoxic capabilities and their ability to modulate immune responses through cytokine secretion (27). In mares, uterine NK (uNK) cells are thought to play dynamic, context-dependent roles during pregnancy, although their functions are less well-characterized than in humans or mice.

Immediately after insemination (Days 0–6), the uterine immune response is dominated by neutrophils and macrophages, which help eliminate excess sperm and potential pathogens. uNK cells appear to play a limited or negligible role during this phase. However, recent studies have identified NK cells in the endometrium of cycling mares, including during diestrus—a progesterone-dominated phase—suggesting a preparatory role for embryo implantation (27). A distinct population of NKp46^+^ CD3^−^ cells has been observed in endometrial biopsies, indicating potential immunomodulatory functions of these cells during the estrous cycle. Their presence in diestrus implies involvement in immune surveillance, tissue remodeling, and the establishment of maternal tolerance processes essential for successful gestation.

As the embryo begins its migration through the uterus (Days 6–16), the maternal immune system transitions into a phase of relative immunological quiescence, minimizing inflammatory responses to support embryonic development. Although direct evidence of uNK cell activity during this phase in mares is lacking, it is hypothesized that these cells may contribute indirectly to maintaining immune tolerance, as seen in other species (2).

The peri-implantation period (Days 20–40) marks a critical window during which uNK cells become increasingly prominent. Single-cell transcriptomic analyses have shown that uNK cells are the most abundant leukocyte population in the equine endometrium during this phase (28). These cells express immunoregulatory markers such as lectin-like receptor (LY49F), CD96, and immunoregulatory receptor with Immunoreceptor Tyrosine-based Inhibitory Motif-ITIM domain (TIGIT), which are associated with maternal–fetal tolerance and modulation of immune responses. Their emergence coincides with the onset of trophoblast invasion and endometrial cup formation, suggesting a role in facilitating vascular remodeling, cytokine balance, and immune adaptation necessary for successful implantation. Although the equine placenta is non-invasive compared to that of humans, the presence of these NK cell subsets indicates conserved mechanisms of immune regulation across species (28).

Between Days 35 and 45, as endometrial cups begin to form, NK-like cells are enriched near the invading trophoblasts. These cells exhibit a non-cytotoxic phenotype and express surface markers including NKp46, CD16, CD56, and CD94. Their localization and phenotype imply a role in modulating local immunity without inducing tissue damage, thereby facilitating the establishment of the semi-allogeneic conceptus (29).

In species such as humans and mice, uNK cells are well recognized for their roles in regulating trophoblast invasion and mediating vascular remodeling—processes essential for successful implantation and placental development (30). These cells interact closely with extravillous trophoblasts (EVTs) and spiral arteries, influencing vascular function and immune tolerance through receptor-ligand mechanisms, such as Killer-cell Immunoglobulin-like Receptors (KIRs) binding to Human Leukocyte Antigen (HLA) HLA-C and HLA-E molecules on EVs.

This supports the hypothesis that uNK cells contribute to placental vascularization and immune tolerance in early pregnancy stages, particularly in humans and mice, and provides a comparative framework for understanding their potential roles in mares.

Although direct functional studies on uNK cells in mares are still lacking, comparative evidence from humans and mice suggests that they may play analogous roles in regulating trophoblast invasion and maintaining immune tolerance. In humans, uNK cells modulate the depth of extravillous trophoblast (EVT) invasion and contribute to spiral artery remodeling processes essential for placental development and maternal–fetal health. These functions are mediated through receptor-ligand interactions, such as Killer-cell Immunoglobulin-like Receptors (KIRs) on uNK cells binding to HLA-C and HLA-E molecules on trophoblasts, which influence cytokine production and immune adaptation (30, 31). By extrapolation, it is hypothesized that equine uNK cells help maintain a delicate balance between sufficient trophoblast penetration and the prevention of excessive invasion, thereby supporting the unique features of equine placentation.

In both humans and mares, immunological tolerance to the trophoblast is essential for successful pregnancy, and uNK cells play a pivotal role in this process. In humans, uNK cells are the most abundant immune population in the decidua during early gestation. They recognize fetal MHC class I molecules, such as HLA-G, and adopt a non-cytotoxic, regulatory phenotype that promotes trophoblast invasion, vascular remodeling, and placental development (32). Similarly, in the mare, NK-like cells have been identified in the endometrium, particularly during the period of endometrial cup formation. Although equine trophoblasts express high levels of paternal MHC-I molecules, they are not targeted by maternal immune cells. This suggests that equine NK-like cells, as described by (33), also assume a specialized, non-cytotoxic role, contributing to immune tolerance and supporting the unique features of equine placentation.

The epitheliochorial placenta, found in species such as horses, cattle, and pigs, represents the least intimate form of placental attachment, characterized by minimal invasion of fetal tissues into the maternal endometrium. This structural feature limits direct immunological interactions at the maternal–fetal interface. While the contribution of pregnancy-associated uterine lymphocytes to successful gestation in epitheliochorial species remains uncertain, current evidence suggests that their activity may be redirected toward promoting fetal survival, even in the absence of deep trophoblast invasion.

The role of uNK cells in species with epitheliochorial placentation, such as the mare, remains highly controversial and poorly understood. Unlike in haemochorial placentation, where uNK cells are abundant and actively involved in trophoblast invasion and vascular remodeling, the structural separation between maternal and fetal tissues in epitheliochorial placentas restricts direct immunological engagement (34). This distinction underscores the importance of placental architecture in shaping immune cell function at the maternal–fetal interface. As highlighted by Sojka (35), comparative studies in mouse and human models reveal significant heterogeneity in uNK cell behavior depending on placental type, suggesting that extrapolations across species must be made with caution.

Building on this hypothesis, further parallels between human and equine pregnancy highlight the central role of uNK cells in establishing immunological tolerance to the trophoblast. While current evidence suggests that equine NK-like cells adopt a non-cytotoxic, regulatory phenotype, further research is needed to characterize their specific markers, activity, and contributions to early pregnancy success.

Table 4 summarizes the current evidence regarding uNK cells in horses, alongside comparative data from other species. Further research is required to elucidate the specific functions of uNK cells across the various stages of equine pregnancy.

**Table 4:** Proposed roles of uterine natural killer (uNK) cells during early pregnancy stages in the mare.

Pregnancy Stage	Proposed Role of uNK Cells	Supporting Evidence
Post-insemination (Days 0–6)	Limited or no direct role; innate immune response dominated by neutrophils and macrophages	No specific uNK cell activity reported; focus is on post-breeding clearance mechanisms (27, 33)
Embryonic Migration (Days 6–16)	Possible immune quiescence: uNK cells may contribute to maintaining tolerance indirectly	No confirmed uNK activity; inflammation during this phase could be detrimental (28)
Peri-implantation (Days 20–40)	Immune regulation and maternal–fetal tolerance; support for vascular remodeling and cytokine balance	uNK cells identified as dominant leukocyte population; express LY49F, CD96, TIGIT (28)
Endometrial Cup Formation (Days 35–45)	Enrichment of NK-like cells near trophoblasts; non-cytotoxic role in modulating local immunity	NKp46, CD16, CD56, CD94 expression elevated in endometrial lymphocytes (29)
Placental Development (Post Day 40)	Hypothesized role in supporting angiogenesis and limiting excessive trophoblast invasion	Comparative inference from humans/mice (30, 31); functional studies in mares still lacking

### 2.2 Transition from a pro-inflammatory to an immune tolerant environment during equine embryo fixation

As the equine conceptus approaches fixation around day 16 of gestation, the uterine environment undergoes a carefully regulated transition from a pro-inflammatory state to one of immune tolerance. This shift is essential to prevent maternal immune rejection of the semi-allogeneic embryo and to facilitate successful implantation and placental development. Hormonal changes during this period—most notably the sustained elevation of P4 secreted by the CL play a central role in directing this immunological adaptation. P4 promotes the expansion of Treg cells and stimulates the production of anti-inflammatory cytokines, including IL-10 and TGF-β, which together act to suppress excessive inflammatory responses and support pregnancy maintenance (2).

Simultaneously, the endometrial microenvironment undergoes localized changes characterized by a reduction in pro-inflammatory mediators such as tumor necrosis factor-alpha (TNF-α) and interleukin-1 beta (IL-1β), along with diminished activation of key inflammatory signaling pathways, including nuclear factor kappa B (NF-κB) and mitogen-activated protein kinase (MAPK). These modifications foster a uterine milieu conducive to the recruitment and activation of tolerogenic DCs, and macrophages, which further reinforce immune tolerance. Progesterone (P4) also contributes to this immunomodulatory landscape by inducing the expression of immune checkpoint molecules, notably Programmed Death-Ligand 1 (PD-L1). PD-L1 is expressed on the surface of various cell types, including immune cells and uterine tissue, and exerts its regulatory function by binding to its receptor, Programmed Death-1 (PD-1), on T cells. This interaction inhibits T cell activation and proliferation, thereby preventing excessive maternal immune responses at the maternal–fetal interface and supporting the establishment of pregnancy (36).

The dynamic interplay between endocrine signals and immune cell populations establishes a localized uterine environment that supports embryo fixation, trophoblast invasion, and placental development, thereby ensuring the maintenance of pregnancy. Disruption of this finely tuned balance—whether due to insufficient P4 signaling, persistent inflammation, or immune dysregulation—can compromise implantation success and increase the risk of EEL (37). Conversely, inadequate immune activation may also result in implantation failure (4). Thus, the coordinated transition from a necessary pro-inflammatory phase to a state of immune tolerance is critical for embryo viability and successful pregnancy in the mare.

Following the initial pro-inflammatory phase essential for conceptus recognition and uterine priming the maternal immune system in the mare appears to shift toward a more balanced or tolerogenic profile, rather than adopting the classical Th2-dominant immune state observed in species with invasive implantation, such as humans and mice (33, 38). This immunological transition is characterized by the recruitment and expansion of Treg cells, identified by the expression of FOXP3, which plays a critical role in suppressing excessive inflammation and promoting maternal tolerance of the semi-allogeneic conceptus (17, 39). While Th2-type cytokines, including IL-4, IL-10, and TGF-β, are likely involved in modulating local immune responses and facilitating tissue remodeling, direct evidence supporting a dominant Th2 environment during early equine pregnancy remains limited (27). In many mammalian species, including humans and rodents, Treg cells, identified by CD4^+^FOXP3^+^ expression, play a central role in this process by suppressing potentially harmful pro-inflammatory immune responses and promoting fetal tolerance Tregs accumulate both systemically and locally at the maternal-fetal interface, secret anti-inflammatory cytokines, such as IL-10 and TGF-β, and engage in direct cell–cell interactions to inhibit effector T cells. Together, these mechanisms create an immune environment conducive to successful implantation and placental development (40).

Anti-inflammatory cytokines, such as IL-10 and TGF-β, play a pivotal role in suppressing pro-inflammatory responses and promoting the expansion of Treg cells, thereby contributing to the prevention of maternal immune rejection of the conceptus (17, 33). IL-15 supports the activation and proliferation of uterine natural killer (uNK) cells, which are essential for endometrial vascular remodeling and the regulation of local immune responses necessary for conceptus fixation (33, 38). Concurrently, molecules such as IFN-γ and granzyme B help modulate innate immune activity, maintaining a controlled cytotoxic environment that safeguards trophoblast integrity while permitting tissue remodeling (14, 38). Additionally, MMPs, secreted by macrophages, facilitate extracellular matrix remodeling and angiogenesis within the endometrium—processes critical for the structural adaptations required during early pregnancy (16, 33). Collectively, these factors coordinate the establishment of a uterine microenvironment that balances immune tolerance with protective immune functions, thereby supporting embryo survival and development in the mare.

Table 5 provides an overview of key immunomodulatory molecules implicated in early equine pregnancy, emphasizing their roles in shaping a balanced uterine environment. These molecules orchestrate immune tolerance, facilitate tissue remodeling, and promote vascular adaptations processes that are critical for conceptus survival and successful implantation in the mare.

**Table 5:** Immunomodulatory molecules involved in early equine pregnancy.

Immunomodulatory Molecules	Function in Early Equine Pregnancy
IL-10	Anti-inflammatory cytokine promotes maternal-fetal tolerance by the suppression of pro-inflammatory responses and supporting Treg expansion
TGF-β	Key regulator of immune tolerance; it induces regulatory T cells (Tregs), inhibits Th1 responses, and supports tissue remodeling in the endometrium
IL-15	Promotes proliferation and activation of uterine NK cells, which participate in vascular remodeling and immune modulation during conceptus fixation
IFN-γ	Produced by NK and T cells; modulates immune surveillance and trophoblast-endometrial interaction; activates innate immune cells and enhances antigen presentation; contributes to conceptus signaling and endometrial receptivity
Granzyme B	Secreted by NK cells; it participates in immune cell-mediated remodeling while maintaining controlled cytotoxicity to protect trophoblasts
MMPs	Secreted by macrophages; degrade extracellular matrix and promote angiogenesis necessary for embryo fixation
PGE2	Produced by the mare’s endometrium, modulates immune response by inhibiting the NK and cytotoxic T cell activation; support corpus luteum maintenance

IL, interleukin; TGF-β, transforming growth factor β; NK, natural killer; IFN-γ, interferon y; MMPs, matrix metalloproteinases; Tregs, regulatory T cells. PGE2, Prostaglandin E2. Source: Data from Figarska and Witkowska-Piłaszewicz (14); Troedsson et al. (14); Dong et al. (16); de Mestre et al. (38); Jaworska et al. (27); Meggyes et al. (36).

While direct phenotypic and functional evidence is still limited, transcriptomic analyses indicate that immune regulatory mechanisms resembling those seen in other species are active during early equine pregnancy. For instance, Castro et al. (41) reported distinct immunomodulatory gene expression patterns in the equine endometrium by days 13 post-ovulation, including markers consistent with Treg activity, such as FOXP3. Additional studies have identified regulatory markers near invasive trophoblast regions and detected Tregs in equine endometrial tissue even prior to conception, suggesting a preconditioned uterine immune environment favoring tolerance (33).

Moreover, mares with lower circulating Treg concentrations have been associated with early embryonic loss before days 40 of gestation (42), supporting the importance of these cells in pregnancy maintenance. Nevertheless, the precise mechanisms by which Tregs mediate immune tolerance in equine pregnancy, including their interactions with cytokines and other immune cells, require further investigation to confirm functional parallels with other species.

As gestation progresses to mid-pregnancy, the mare’s immune environment shifts further toward an anti-inflammatory profile. This is evidenced by downregulation of Th1-associated transcripts and upregulation of Th2- and Treg-related gene expression, coinciding with increased concentrations of IL-10, TGF-β, and IL-4. Such a balanced immune state appears essential to dampen cytotoxic T cell activity and regulate uN cell function, thereby promoting placental development and fetal growth (18). This temporal regulation of immune responses aligns broadly with observations in other mammalian species but requires equine-specific functional studies for a comprehensive understanding. Other anti-inflammatory Th2-type cytokines such as IL-5 and IL-13 also have been implicated in promoting a Th2-skewed immune environment that suppresses cytotoxic Th1 responses, thereby supporting embryo survival and facilitating the tissue remodeling necessary for implantation (27). *In vitro* studies have demonstrated that IL-4 modulates equine endometrial cell function, providing direct evidence of its immunoregulatory potential in the mare (43). Elevated uterine and systemic levels of IL-5 and IL-10 have been correlated with successful pregnancies, whereas increased concentrations of pro-inflammatory cytokines, including IL-2 and TNF-α have been linked to pregnancy complications such as placentitis (44).

An emerging area of interest in equine reproductive immunology is the potential involvement of transthyretin (TTR), a transport protein traditionally known for carrying thyroid hormones and retinol. A relationship between thyroid function and seasonal reproductive activity was investigated in mares (45, 46) and in donkeys (47), confirming the active metabolic role of these hormones in the first trimester of pregnancy, contributing to the control of early embryonic growth and development, before the onset of fetal thyroid activity (48). Recent studies suggest that TTR may also participate in modulating uterine immune responses under conditions of stress or infection, as elevated TTR concentrations have been detected in uterine fluid of mares subjected to infection or corticosteroid treatment (1, 49). TTR’s capacity to cleave apolipoprotein A1 and subsequently inhibit the pro-inflammatory cytokine IL-1β hints at a regulatory role during inflammatory uterine conditions. However, its involvement during normal equine pregnancy remains speculative and warrants further investigation.

In the mare, although specific characterization of tolerogenic antigen-presenting cells (APCs) in the endometrium during gestation remains limited, transcriptomic and phenotypic evidence suggests the presence of cell populations with immunomodulatory functions consistent with a tolerogenic profile (33, 41). These cells contribute to creating a uterine microenvironment that promotes tolerance toward the semi-allogeneic embryo, regulating the maternal immune response to prevent conceptus rejection. In other mammalian species, such as humans and rodents, tolerogenic APCs, particularly certain subpopulations of uterine DCs and macrophages, play a key role in the induction and maintenance of maternal-fetal tolerance. These cells promote the expansion of Treg cells through secretion of anti-inflammatory cytokines, like IL-10 and TGF-β, and controlled presentation of fetal antigens, thereby facilitating implantation and placental development (50). The presence of analogous mechanisms in the mare suggests evolutionary conservation of these immunological strategies, although more detailed functional studies are needed to confirm the precise role of tolerogenic APCs in equine pregnancy.

The maternal immune system undergoes complex adaptations during early pregnancy to promote tolerance toward the semi-allogeneic embryo while maintaining defense mechanisms. Key immune components, as regulatory T cells, anti-inflammatory cytokines, and tolerogenic APCs, appear to play crucial roles in modulating uterine immunity in mares, like other mammalian species. Although direct functional evidence in equines remains limited, transcriptomic and immunohistochemical studies suggest conserved mechanisms that support embryo implantation and placental development.

Table 6 summarizes the primary immune mediators implicated in early equine pregnancy, highlighting their roles, current evidence in the mare and comparative insights from other species.

**Table 6:** Principal immune mediators involved in early equine pregnancy: functional roles, supporting evidence, and comparative insights across species.

Immune Components	Role in Early Pregnancy	Evidence in Mare	Comparative Notes	Key References
Regulatory T Cells (Tregs)	Suppress pro-inflammatory responses, promote fetal tolerance via IL-10, TGF-β secretion, cell–cell contact	FOXP3 expression detected in endometrium by day 13 post-ovulation; presence noted pre-conception; low Treg levels associated with embryonic loss	Central role is established in humans, rodents; functional data in mares still limited	(14, 17, 27, 33, 38)
Anti-inflammatory Cytokines	Promote Th2 dominant environment, suppress Th1 cytotoxicity, support the implantation and tissue remodeling	IL-4 modulates the equine endometrial cells; IL-5, IL-10 concentrations correlate with pregnancy success; IL-2 and TNF-α are linked to complications	Th2 cytokines are critical in multiple species; *in vitro* evidence supports immunoregulatory role in mares	(2, 17, 18, 22, 24, 33)
Tolerogenic Antigen-Presenting Cells (APCs)	Induce maternal-fetal tolerance by expanding Tregs, secreting IL-10 and TGF-β, and presenting fetal antigens	Transcriptomic and phenotypic markers suggest tolerogenic APC populations in endometrium; limited functional data	Well characterized in humans and rodents, less understood in mares	(33, 41, 50)
Transthyretin (TTR)	Potential modulator of uterine immune response during stress or infection by inhibiting IL-1β	It is elevated in uterine fluid during infection/corticosteroid treatment; its role in normal pregnancy is unknown	Novel hypothesis; no established role in equine pregnancy	(45–49)
Immune Shift to Anti-inflammatory Profile (mid-pregnancy)	Downregulation of Th1 markers; upregulation of Th2 and Treg markers; increased IL-10, TGF-β, and IL-4	Transcriptomic data shows immune shifts in mares coinciding with placental development	Similar temporal immune regulation described in other mammals	(39, 40)

### 2.3 Molecular mediators of early implantation in the mare

Between the 3rd and 4th week of pregnancy in mares, genes encoding cytokines, growth factors, hormones, and their receptors are highly expressed in the trophectoderm, endometrium, or both reflecting the dynamic molecular interactions that underpin early gestational development (51, 52).

Recent transcriptomic analyses have revealed the upregulation of various cytokines, growth factors, and corresponding receptors in equine trophectoderm and endometrium during early gestation. This molecular upregulation is driven by luteal-derived P4 and locally produced conceptus hormones, such as estrogens and PGE2, underscoring the dynamic crosstalk between maternal and embryonic tissues (52, 53). Among the signaling molecules implicated in this process, many of which are well established in implantation biology across mammalian species, are LIF, OPN, integrins, and members of the insulin-like growth factor (IGF) and fibroblast growth factor (FGF) families (54–56). LIF has emerged as a key mediator of early implantation in the mare, facilitating molecular communication between the conceptus and the maternal endometrium. The expression of LIF and its receptors (LIFR and gp130) increase markedly around days 21 of gestation, peaking by days 28, coinciding with the development of a receptive uterine environment (55).

Similarly, OPN expressed by the uterine epithelium, is involved in conceptus attachment through its interaction with integrins, which mediate essential cell–cell and cell–matrix adhesion events Integrins not only anchor the conceptus to the uterine wall but also transduce intracellular signals necessary for implantation success (57).

Additionally, growth factors such as IGFs and FGFs, key regulators of cell proliferation, differentiation, and tissue remodeling, are dynamically expressed in the equine endometrium during early gestation. Insulin-like Growth Factor Binding Protein 1 (IGFBP1), for example, is expressed in the endometrium between days 8 and 13.5 of gestation, supporting conceptus–endometrial communication and modulation of trophoblast invasion (56). Similarly, Fibroblast Growth Factor 9 (FGF9) and Tissue Inhibitor of Metalloproteinases 1 (TIMP1) are regulated in the preimplantation endometrium, contributing to endometrial proliferation and tissue remodeling (56). Single-cell RNA sequencing studies have further identified distinct endometrial subpopulations before and after implantation, showing regulation of genes associated with cellular homeostasis, metabolism, and fibrosis, mediated by cytokines, such as TGF-β1, IL-4, IL-13, and IL-17 which contribute to uterine receptivity and the endometrial adaptations required for successful implantation (58).

A comprehensive transcriptomic study by Ulaangerel et al. (59) examined the equine endometrium during the critical period of embryo fixation, between days 16 and 22 of gestation. This period is essential for establishing maternal–embryo communication and ensuring implantation success. The authors identified significant changes in gene expression related to immune regulation, cell signaling, ECM remodeling, and hormone responsiveness. Notably, they observed upregulation of anti-inflammatory cytokines, such as TGF-β1 and IL-10, suggesting that the endometrium shifts towards a tolerogenic environment that supports the semi-allogeneic embryo. The study also highlighted increased expression of key molecular mediators of implantation, including LIF and its receptors, as well as adhesion molecules such as integrins and OPN (60). These factors play crucial roles in facilitating embryo adhesion and enhancing endometrial receptivity. Genes involved in tissue remodeling, such as MMPs and their inhibitors (TIMPs), were dynamically regulated, reflecting the active restructuring of the endometrial ECM necessary to accommodate trophoblast invasion and conceptus expansion. Elevated expression of growth factors, including FGFs and IGFs, further indicated their role in promoting cell proliferation and supporting early embryonic development (59). Overall, this research provides valuable molecular insights into how the equine endometrium adapts during early pregnancy, highlighting potential biomarkers for uterine receptivity and identifying targets that could be leveraged to improve reproductive success in mares. Nevertheless, despite these advances, the precise mechanisms by which the equine endometrial surface accommodates the dual implantation strategies the localized invasion by chorionic girdle cells versus the interdigitated apposition by non-invasive chorionic cells remain poorly understood and warrant further investigation.

In support of this, recent single-cell transcriptomic analysis of the equine endometrium has revealed active immune regulation during implantation, despite its delayed and non-invasive nature. Sixteen distinct immune cell clusters were identified, with natural killer (NK) cells being the most abundant. These cells expressed genes associated with fetal MHC I interaction and immunoregulatory pathways, including CD96 and TIGIT (T cell immunoreceptor with Ig and ITIM domains). Additionally, the expression of C-X-C chemokine receptor type 4 (CXCR4) across multiple immune subtypes suggests conserved mechanisms of embryo–maternal communication. These findings indicate that, although equine implantation differs structurally and temporally from more invasive models, it shares key immunological features with other species (28).

Table 7 summarizes key molecular mediators involved in early implantation in the mare, highlighting their functional roles, timing and location of expression, and relevant supporting references. These factors include cytokines, growth factors, hormones, and adhesion molecules that coordinate the complex interaction between the equine conceptus and maternal endometrium during the critical stages of embryo fixation and uterine receptivity. The coordinated action of these molecules facilitates endometrial adaptation, conceptus adhesion, and immune modulation necessary for successful pregnancy establishment.

**Table 7:** Molecular mediators of early implantation in the mare, detailing their roles, temporal and spatial expression.

Molecular Mediators	Expression Timing	Location	Role/Function
Leukemia Inhibitory Factor (LIF)	Peaks around days 21–28	Endometrium and Trophectoderm	Facilitates conceptus-endometrium communication; promotes trophoblast adhesion and uterine receptivity
Osteopontin (OPN)	Early gestation	Uterine epithelium	Mediates conceptus attachment via integrin interaction
Integrins	Early gestation	Endometrium	Anchor the conceptus to the uterine wall; involved in cell adhesion and intracellular signaling
IGFBP1 (IGF binding protein 1)	Days 8–13.5	Endometrium	Modulates trophoblast invasion; enhances conceptus–endometrial interaction
FGF9 (Fibroblast Growth Factor 9)	Preimplantation	Endometrium	Regulates stromal cell proliferation and endometrial tissue remodeling
TIMP1 (Tissue Inhibitor of Metalloproteinases 1)	Preimplantation	Endometrium	Inhibits MMPs; controls extracellular matrix degradation and remodeling
TGF-β1, IL-4, IL-13, IL-17	Before and after implantation	Endometrium	Regulate cellular homeostasis, metabolism, fibrosis; contribute to uterine receptivity
IL-10	Days 16–22 (embryo fixation period)	Endometrium	Anti-inflammatory cytokine; promotes maternal immune tolerance to the conceptus
MMPs and TIMPs	Early gestation	Endometrium	Coordinate extracellular matrix remodeling and regulate trophoblast invasion dynamics

Source: Data from De Ruijter-Villani et al. (55); Gibson et al. (56); Drzewiecka et al. (58); Ulaangerel et al. (59); Johnson et al. (60).

The maternal immune system undergoes complex adaptations during early pregnancy to establish a tolerant environment that supports embryo implantation and fetal development. Table 8 summarizes the key immune cell populations, cytokines, and molecular mediators involved in these processes, highlighting their dynamic roles and the current state of knowledge based on recent studies in the mare. The cited literature supports the immune functions and adaptations outlined in this work. Despite the general conservation of immune processes among mammals, a deeper understanding of the mare’s distinctive immunological strategies during early pregnancy remains a priority for future research.

**Table 8:** Principal immune cell subsets, cytokines, and molecular mediators driving immunological adaptations in early equine pregnancy.

Immune Components	Changes/role during early pregnancy	References
Innate Immunity		
Neutrophils and Macrophages	Recruited to endometrium; contribute to transient pro-inflammatory phase, supporting implantation and conceptus mobility.	(9–11, 76)
Dendritic Cells (Tolerogenic APCs)	Activated by seminal plasma; promote tolerogenic environment; induce expansion of Tregs via IL-10 and TGF-β secretion; regulate maternal immune tolerance.	(17, 18, 33, 41, 50)
Uterine NK (uNK) cells	Display non-cytotoxic phenotype; support vascular remodeling and local immune tolerance.	(22, 33, 38, 54)
Adaptive Immunity		
Regulatory T Cells (Tregs)	Expand locally and systemically; secrete IL-10 and TGF-β; suppress pro-inflammatory responses and promote fetal tolerance.	(14, 17, 27, 33, 38)
Th1/Th2 Cytokine Balance	Shift toward Th2 (IL-4, IL-5, IL-13) cytokines, reducing Th1 cytotoxicity; facilitates immune tolerance and tissue remodeling.	(18, 27, 43, 44)
Anti-inflammatory Cytokines	IL-10, TGF-β, and others upregulated to promote tolerogenic environment; they are crucial for implantation and placental development.	(11, 18, 24, 50, 59)
Additional Immune Factors		
Transthyretin (TTR)	Potential immunomodulatory role; may inhibit pro-inflammatory cytokines during uterine stress/infection; its role in normal pregnancy unclear.	(45, 46)

### 2.4 Endometrial cups and maternal immune response in the mare

Endometrial cups (ECs) are specialized, transient structures unique to equine pregnancy. They arise from invasive trophoblast cells of the chorionic girdle, initiating development between days 35 and 40 of gestation. These trophoblasts penetrate the uterine epithelium and embed within the endometrial stroma—an uncommon feature for the typically non-invasive epitheliochorial placentation in horses, and one that shares certain characteristics with the hemochorial placentation found in primates and rodents (61). Histologically, the ECs become visible around days 38, peak in size and secretory function near days 60, and regress between days 100 and 120 of gestation (62). Their primary function is the secretion of equine chorionic gonadotropin (eCG), a hormone essential for the formation of secondary corpora lutea and maintaining P4 concentrations in early pregnancy. This luteotrophic function supports pregnancy maintenance (1, 61).

Notably, ECs are of fetal origin and express paternal MHC antigens, rendering them semi-allogeneic to the maternal immune system. This semi-allogenicity poses a challenge for immune tolerance, requiring a finely tuned local immune environment to prevent maternal rejection. The trophoblasts express a restricted and specialized set of classical and non-classical MHC class I molecules with low polymorphism, which minimizes direct recognition by maternal cytotoxic lymphocytes while enabling immunomodulatory interactions (63).

Maternal immune tolerance toward the ECs is initially sustained by a locally immunosuppressive microenvironment, characterized by an enrichment of regulatory T cells (Tregs; CD4^+^CD25^+^FOXP3^+^) and elevated levels of anti-inflammatory cytokines, such as IL-10 and TGF-β. Additional immunomodulatory mechanisms, including the expression of indoleamine 2,3-dioxygenase (IDO) and immune checkpoint molecules like programmed death-ligand 1 (PD-L1) by trophoblast cells, further suppress effector T cell activation, thereby protecting the ECs from maternal cytotoxic responses (18).

As gestation advances, this immunological balance gradually shifts from tolerance toward immune recognition and targeting. Increased infiltration of cytotoxic T lymphocytes, macrophages, and NK cells is observed at the cup sites, coinciding with the upregulation of classical MHC class I molecules by trophoblasts, which enhances their immunogenic visibility. Concurrently, pro-inflammatory cytokines such as IFN-γ and TNF-α become elevated, promoting immune cell recruitment and contributing to the immune-mediated regression of the ECs (38, 62).

Recent single-cell RNA sequencing (scRNA-seq) and spatial transcriptomic analyses have revealed complex, spatially organized immune populations at the maternal–fetal interface. These include CD8^+^ cytotoxic T cells, CD4^+^ helper T cells, and regulatory T cells (Tregs), which collectively maintain the immune balance required for trophoblast survival and the eventual regression of ECs (62). Innate immune cells such as macrophages, DCs, and eosinophils also contribute to both immune modulation and structural remodeling of the endometrial stroma (2). The local cytokine milieu, enriched in IL-10, TGF-β, and IDO, supports trophoblast survival by suppressing maternal immune activation. The restricted MHC expression and immunomodulatory molecule production by trophoblasts effectively reduce the maternal recognition of paternal antigens early on, but this immune privilege is transient. The controlled maternal immune response to these paternal antigens ultimately leads to apoptosis of trophoblast cells and cup regression between days 100 and 120 of gestation.

Understanding the biology of ECs and the immune dynamics associated with paternal antigen recognition is essential for advancing equine reproductive health, particularly in the context of immune-mediated EEL or pregnancy failure (62). Furthermore, the equine model offers valuable comparative insights into mechanisms of immune tolerance relevant to human reproductive immunology and transplantation biology (61). Emerging techniques, such as spatial transcriptomics and epigenetic profiling, continue to deepen knowledge of immune-trophoblast interactions at the maternal-fetal interface, paving the way for potential immunomodulatory therapies to improve pregnancy outcomes (62).

Overall, the cytokine environment at the ECs sites reflects a Th2-skewed immune profile, characterized by elevated anti-inflammatory cytokines such as IL-10 and TGF-β. This Th2 dominance promotes immune tolerance, facilitates controlled trophoblast invasion, and supports endometrial remodeling. In contrast, Th17 cell involvement appears minimal, aligning with the need to limit pro-inflammatory responses that could compromise pregnancy maintenance (18).

Table 9 summarizes the key stages of EC development and regression, highlighting the associated immune cell dynamics and hormonal changes throughout gestation.

**Table 9:** Immune tolerance and activation at the endometrial cups: a critical balance for the establishment and maintenance of pregnancy in mares.

Gestational Days	Events	Immune Changes	Hormonal Changes
35–40	Initial formation of endometrial cups from invasive trophoblast cells of the chorionic girdle.	Early immune tolerance mediated by increased regulatory T cells (Tregs) and anti-inflammatory cytokines (IL-10, TGF-β). Expression of paternal MHC antigens by trophoblasts, requiring immune modulation.	Beginning of eCG secretion.
36–38	Origin of invasive trophoblast cells that form endometrial cups.	Local immunosuppressive microenvironment was established to protect trophoblasts from maternal immune attack, including expression of IDO and immune checkpoint molecules.	eCG secretion begins to rise.
~38	Endometrial cups become histologically visible in the endometrium.	Continued immune modulation with dominance of Tregs and innate immune cells (macrophages, dendritic cells).	Increasing eCG production supports luteal function.
60	Peak size and secretory activity of endometrial cups (eCG production).	Immune tolerance maintained; low cytotoxic T cell activity; anti-inflammatory cytokines high.	Maximum eCG concentrations; maintenance of secondary corpora lutea and P4.
60–100	Active secretion of eCG to support secondary corpora lutea and P4 maintenance.	Gradual infiltration of cytotoxic T lymphocytes (CTLs), macrophages, and NK cells; beginning of immune recognition of paternal antigens.	Sustained high P4 via luteal support from eCG.
100–120	Immune-mediated regression and sloughing of the endometrial cups.	Increased pro-inflammatory cytokines (IFN-γ, TNF-α); infiltration of activated CD8 + T cells and neutrophils; apoptotic trophoblast.	Decreased eCG secretion; P4 concentrations eventually decline as cups regress.

Source: Data from de Mestre et al. (38); Fedorka and Ball (61). Rapacz-Leonard et al. (63).

## 3 Mid-gestation (days 40–250)

As gestation advances, the uterine environment undergoes a shift toward an anti-inflammatory and immunotolerant state, essential for proper placental function and fetal development. This transition involves elevated levels of IL-10 and TGF-β, which help suppress pro-inflammatory activity and facilitate maternal tolerance to the semi-allogeneic fetus (18, 33). Maintaining this delicate immunological equilibrium not only prevents excessive immune activation but also supports the structural remodeling required for placental expansion.

**Figure 1:**
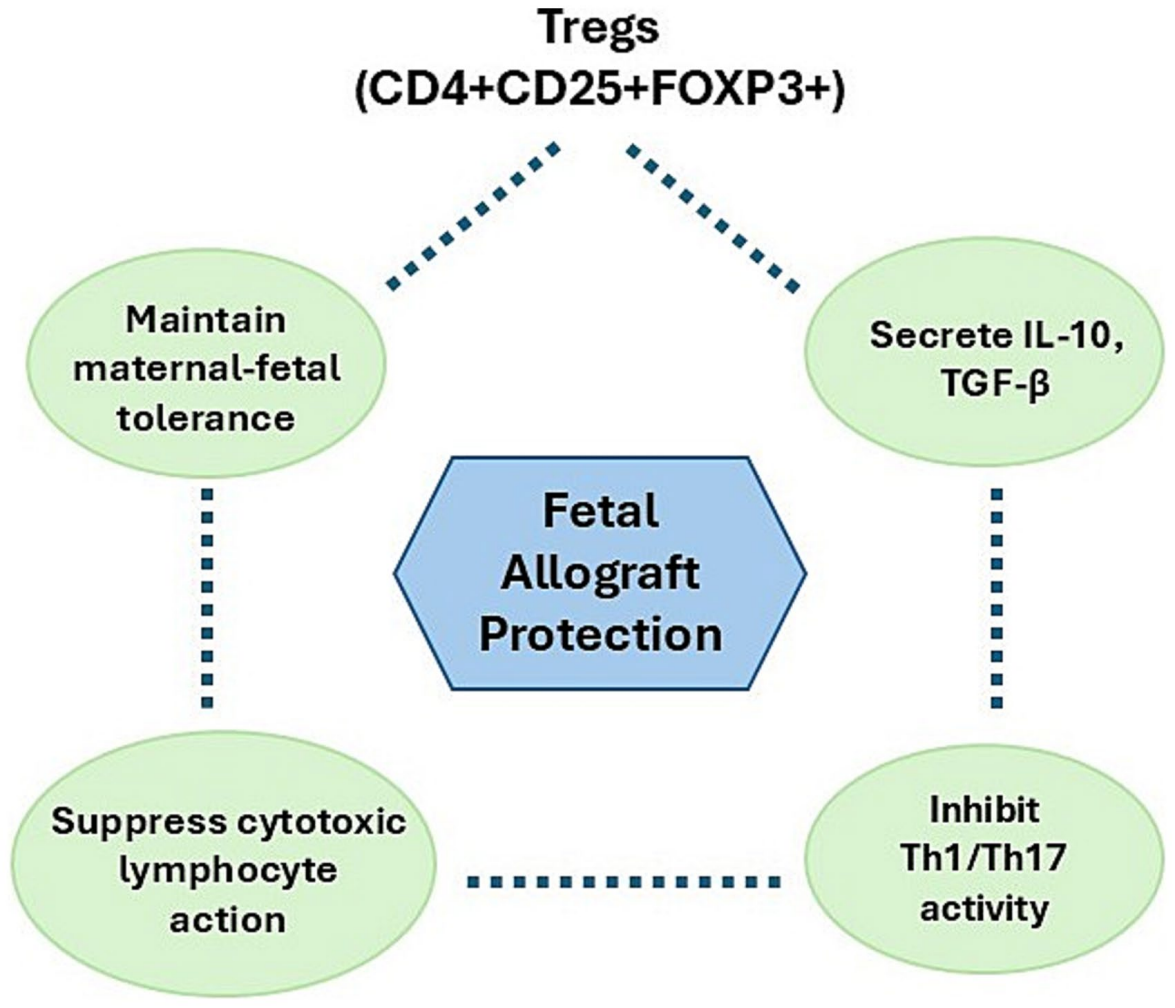
Treg-mediated immune modulation in equine mid-gestation.

In accordance with this immunological shift, pregnant mares do not exhibit significant changes in the concentrations of IL-1β, IL-2, IL-4, IL-17, IFN-γ, or TNF-α compared to non-pregnant controls. However, levels of Interleukin-1 Receptor Antagonist (IL-1RA) and IL-10 peak around mid-gestation—particularly during the sixth month before declining as term approach (64). At this stage, the immune profile is characterized by a predominance of Th2 cytokines and heightened regulatory T cell (Treg) activity, which collectively suppress cytotoxic lymphocyte responses at the maternal-fetal interface (18, 38). Supporting this, Figarska et al. (64) demonstrated that the “Th2 phenomenon” observed in human pregnancy also occurs in mares. This phenomenon entails a shift from pro-inflammatory Th1 cytokines (IL-1β, IL-2, IFN-γ) toward anti-inflammatory Th2 cytokines (IL-4), fostering an immunological environment favorable to fetal tolerance (Figure 1).

Additionally, factors such as IGFs, fibroblast growth factors (FGFs), and TIMPs contribute to placental vascular development, fetal development, thereby preserving uterine integrity (14, 33). These adaptations promote maternal immune tolerance, regulate trophoblast invasion, and support placental function—all of which are crucial for fetal development and the maintenance of pregnancy (4, 18, 33, 44). However, further research is needed to clarify the specific roles of these mediators in the mare.

Table 10 summarizes the key changes in immune mediators and highlights their roles in promoting maternal tolerance, regulating trophoblast invasion, and supporting placental function—processes essential for fetal development and the successful maintenance of pregnancy.

**Table 10:** Principal immune adaptations linked to a Th2-dominant profile during mid-gestation in mares.

Immune Components/Mediators	Changes during Mid-Gestation	Functions/Significance
Upregulation of Th2 cytokines (e.g., IL-4, IL-5, IL-10)	↑ Upregulated	Promote anti-inflammatory responses; enhance maternal tolerance
TGF-β	↑ increased expression	Suppresses pro-inflammatory activity; supports tissue remodeling and immune regulation
Regulatory T Cells (Treg)	↑ enhanced number and activity	Inhibit cytotoxic T cells and NK cell function; maintain fetal tolerance and vascular remodeling
Cytotoxic T cells/NK cells	↓ suppressed activity	Reduced cytotoxicity protects fetal tissues from immune attack
Pro-inflammatory cytokines (e.g., IFN-γ, TNF-α)	↓ Downregulated or modulated by Th2/Treg dominance	Prevent excessive inflammation that could compromise pregnancy
Growth Factors (IGF and FGF)	↑ Upregulated	Support placental growth, angiogenesis, and fetal development
Tissue Inhibitors of Metalloproteinases (TIMP)	↑ Increased concentrations	Regulate trophoblast invasion; preserve uterine structure and prevent excessive ECM degradation

Source: Data from Christoffersen and Troedsson (4); Troedsson et al. (14); Fedorka et al. (18); Jaworska et al. (33); Fedorka et al. (44); Figarska et al. (64).

Transcriptomic studies of the equine endometrium and chorioallantois have uncovered dynamic shifts in immune gene expression throughout gestation. RNA sequencing reveals a progressive upregulation of genes associated with regulatory T cells (Tregs) during pregnancy, in contrast to the diestrus endometrium. These genes peak between Days 45 and 180, then decline prior to parturition (65). This transcriptional profile reflects the emergence of an immune environment dominated by Th2 cytokines and Tregs, beginning as early as Days 45–120, which plays a critical role in placental development and fetal survival (18).

A hallmark of equine pregnancy is the tightly regulated temporal and spatial expression of MHC molecules. Most trophoblast cells of the allantochorion lack surface MHC class I proteins, likely to avoid triggering maternal immune responses similar to what is observed in cattle (66). In contrast, trophoblasts of the chorionic girdle and ECs transiently express high levels of polymorphic MHC class I antigens derived from both maternal and paternal sources during early placentation (22). This distinctive pattern, uncommon in other species, may facilitate localized immune modulation, supported by decidual immune cells and Tregs that help suppress potentially harmful maternal reactions.

Functional studies further reinforce the importance of immune tolerance mechanisms in the mare. During pregnancy, maternal peripheral blood lymphocytes exhibit reduced cytotoxic T cell activity against paternal antigens, a suppression that reverses postpartum. This dampened immune response may be driven by trophoblast-derived soluble factors that inhibit lymphocyte proliferation and cytokine production (67). Moreover, endometrial lymphocytes within the cups express NK cell markers, including NKp46, CD16, CD56, and CD94 at levels higher than in peripheral blood, suggesting specialized uterine NK cell roles in supporting maternal-fetal tolerance (35). In any case, any local inflammatory process does not appear to be reflected at the systemic level, as the profiles of acute phase proteins including serum amyloid A and C-reactive protein remain unchanged during this period in the mare (68).

## 4 Immunological and inflammatory changes during late equine gestation and parturition (days 250–340)

In the final stages of equine gestation, the maternal immune system and uterine environment undergo pivotal changes to initiate parturition. This transition is marked by localized activation of inflammatory pathways within the uterus and cervix, which are essential for tissue remodeling and the onset of labor. The diffuse epitheliochorial placenta, characterized by six distinct tissue layers separating maternal and fetal blood supplies, enables tightly regulated immune interactions during placentation. These interactions are mediated through the formation of microcotyledons by allantochorionic villi, which facilitate nutrient exchange while maintaining immunological separation (61, 69).

### 4.1 Key molecular and immune mediators

Late gestation and the onset of parturition are marked by a pronounced increase in pro-inflammatory cytokines within both the endometrial and myometrial compartments. Among these, IL-6 plays a dual role by activating innate immune responses and modulating physiological stress associated with labor. Interleukin-8 (IL-8), meanwhile, facilitates the recruitment of neutrophils, which are essential for cervical remodeling and the initiation of myometrial contractions (33). In the final month of pregnancy, elevated levels of IL-17 and TNF-α further prime the uterus for labor by enhancing local inflammatory signaling (64).

A transcriptomic study by El-Sheikh et al. (70). analyzed gene expression in the equine chorioallantois during labor and identified 4,137 differentially expressed genes 1,820 upregulated and 2,317 downregulated compared to preterm, non-laboring tissue. Labor was associated with increased expression of proinflammatory mediators, including MHC class I and II molecules, NLRP3, CXCL8, macrophage migration inhibitory factor (MIF), and several matrix metalloproteinases (MMP1, MMP2, MMP3, MMP9). Apoptosis-related genes such as ATF3, ATF4, FAS, FOS, and BIRC3 were also upregulated, indicating active inflammation, ECM degradation, and programmed cell death. Simultaneously, 21 collagen transcripts were downregulated, suggesting structural weakening of the fetal membranes. These molecular events likely contribute to placental separation, a process further facilitated by MMPs secreted by neutrophils, macrophages, and endometrial cells, which degrade ECM components. Following rupture of the umbilical cord, ischemia in placental vessels may promote contraction of the allantochorion and loosening of fetal-maternal attachments. Coordinated uterine contractions then complete the delivery process, ensuring successful parturition.

Chemokines such as CCL2 (monocyte chemoattractant protein-1, MCP-1) and CXCL10 contribute to immune cell infiltration by attracting monocytes, macrophages, T lymphocytes, and NK cells. This immune cell influx supports tissue remodeling and functional changes necessary for labor progression. Simultaneously, rising concentrations of PGF2α stimulate myometrial contractility and induce luteolysis, leading to a decline in P4 levels and the initiation of labor (71).

### 4.2 Immune profile dynamics, cellular activation, and key inflammatory mediators

As pregnancy approaches term, the immune environment undergoes a marked transition from an anti-inflammatory, Th2-dominant profile to a pro-inflammatory, Th1-skewed state. Elevated levels of IFN-γ and TNF-α activate immune pathways that promote placental detachment and initiate labor (72). Concurrently, MMPs released by neutrophils, macrophages, leukocytes, and endometrial cells contribute to the degradation of ECM components including collagens, fibronectin, elastin, and proteoglycans facilitating cervical ripening, placental separation, and effective uterine contractions (33).

Toward the end of gestation, levels of nitric oxide (NO) and PGE2 increase significantly. NO facilitates vasodilation and smooth muscle relaxation, while PGE2 plays a critical role in cervical softening and dilation (73). This tightly regulated inflammatory cascade coordinates cervical remodeling, stimulates uterine contractions, and recruits immune cells for postpartum uterine recovery. Any disruption in this process may lead to complications such as prolonged labor, retention of fetal membranes, and increased susceptibility to infections, all of which can negatively impact the mare’s health (72).

## 5 Immune and molecular changes during postpartum in mares

Following parturition, the mare’s reproductive tract undergoes substantial remodeling and physiological recovery, driven by distinct immunological and molecular shifts that are critical for uterine involution and the restoration of fertility. This postpartum phase is marked by a transient yet tightly regulated inflammatory response, designed to eliminate residual tissue and prevent infection. Among the key changes observed:

- Upregulation of pro-inflammatory cytokines: In the immediate postpartum period, local uterine levels of cytokines such as interleukin-1β (IL-1β), IL-6, IL-8, and TNF-α rise significantly. These mediators promote the recruitment and activation of neutrophils and macrophages, which are essential for the clearance of fetal membranes, microbial pathogens, and necrotic debris (33, 70).
- Neutrophil influx and phagocytic activity: Neutrophils act as primary responders in the postpartum uterus, swiftly migrating to the site to engulf and eliminate bacteria and cellular debris. Their phagocytic activity is essential for mitigating the risk of postpartum uterine infections, including endometritis (74).
- Transition to an anti-inflammatory milieu: Following the initial pro-inflammatory phase, the uterine environment shifts toward a more anti-inflammatory profile. Cytokines such as IL-10 and TGF-β become predominant, facilitating the resolution of inflammation and supporting tissue repair and regeneration (72).
- Role of matrix metalloproteinases (MMPs): MMPs play a pivotal role in remodeling the extracellular matrix during uterine involution, contributing to the restoration of normal tissue architecture and function (75).
- Hormonal modulation: The sharp postpartum decline in P4 levels, coupled with a rise in estrogen, exerts significant influence on immune cell activity and enhances uterine contractility, thereby promoting efficient tissue repair and recovery.

Precise regulation of the immune and molecular processes involved in postpartum uterine recovery is essential to avoid complications such as retention of fetal membranes, delayed involution, and uterine infections all of which can compromise reproductive efficiency and long-term fertility in mares (70).

This review emphasizes the central role of finely regulated immune and inflammatory processes throughout equine pregnancy and parturition. In early gestation, a localized pro-inflammatory response driven by cytokines such as IL-1β, TNF-α, IL-6, IL-8, and IFN-γ facilitates embryo implantation, endometrial receptivity, and trophoblast invasion through immune cell recruitment and vascular remodeling. During this phase, the formation of endometrial cups, specialized structures derived from fetal trophoblast cells, plays a key role in modulating maternal immune tolerance by secreting eCG, which supports luteal function and P4 production. As gestation progresses, the maternal immune environment transitions to a Th2-dominant, anti-inflammatory state, essential for maintaining maternal-fetal tolerance and supporting fetal development. Key cytokines including IL-4, IL-5, IL-10, IL-13, and TGF-β suppress inflammatory activity and promote tissue remodeling, creating a stable uterine environment that prevents immune rejection. In late gestation, the immune profile shifts back toward a Th1-skewed inflammatory state, marked by increased levels of IFN-γ, TNF-α, IL-1β, IL-6, and IL-8, which activate pathways necessary for placental separation, cervical ripening, and uterine contractions. These cytokines act in concert with rising concentrations of NO and PGE2, facilitating smooth muscle activation and extracellular matrix degradation. Following parturition, a transient inflammatory response supports uterine clearance and initiates tissue repair. Altogether, these dynamic immune adaptations including the contribution of ECs are essential to the physiological establishment, progression of pregnancy, initiation of labor, and postpartum recovery in mares.

## 6 Conclusion

Immune and inflammatory processes are central to equine pregnancy, orchestrating each stage of fetal development and ensuring reproductive success in the mare. Early gestation is marked by a controlled pro-inflammatory response that facilitates embryo mobility and implantation. This swiftly transitions into a tolerogenic immune environment, allowing the semi-allogeneic conceptus to be accepted and supported. During mid-gestation, finely tuned immune modulation and the predominance of anti-inflammatory cytokines maintain a delicate balance—protecting the fetus while preserving maternal immune surveillance. As parturition approaches, a resurgence of pro-inflammatory signals becomes essential to initiate labor, highlighting that inflammation is not merely a pathological response but a vital physiological mechanism in equine reproduction. This dynamic and tightly regulated immunological sequence underscores the critical role of immune processes in maintaining pregnancy and preparing for delivery.

In-depth, multidisciplinary research into these immune mechanisms is essential—not only to enhance reproductive strategies in mares, but also to prevent pregnancy-related complications and ensure the well-being of both mare and foal. Recognizing the central role of immune regulation in equine gestation is fundamental to progress in reproductive equine medicine.

## Glossary

3NLRP3: NOD-like receptor pyrin domain-containing
APC: Antigen-Presenting Cell
ATF: Activating Transcription Factor
BIRC 3: Baculoviral IAP Repeat Containing 3
CCL2: Chemokine (C-C motif) ligand 2 (Monocyte Chemoattractant Protein-1, MCP-1)
CCL5/RANTES: Chemokine (C-C motif) ligand 5/Regulated on Activation, Normal T Cell Expressed and Secreted
CD38: Cluster of Differentiation 38
CD4: Cluster of Differentiation 4
CD4^+^FOXP3^+^ Tregs: CD4 positive FOXP3-expressing Regulatory T cells
CD8 + T cells: CD8 positive Cytotoxic T cells
CL: Corpus Luteum
CRISP3: Cysteine rich secretory protein 3
CTL: Cytotoxic T lymphocytes
CXCL10: Chemokine (C-X-C motif) ligand 10
CXCL8: C-X-C Motif Chemokine Ligand 8 (also known as IL-8)
DCs: Dendritic Cells
EC: Endometrial Cups
eCG: Equine Chorionic Gonadotropin
ECM: Extracellular matrix
EEL: Early embryonic loss
EVT: Extra villous trophoblast
FAS: Fas Cell Surface Death Receptor
FGF: Fibroblast Growth Factor
FGF9: Fibroblast Growth Factor 9
FGFs: Fibroblast Growth Factors
FOS: Fos Proto-Oncogene, AP-1 Transcription Factor Subunit
FOXP3: Forkhead box P3 (transcription factor characteristic of regulatory T cells)
GFs: Growth Factors
Granzyme B: Cytotoxic protein secreted by NK cells
IDO: Indoleamine 2,3-dioxygenase
IFN-γ: Interferon Gamma
IGF: Insulin-like Growth Factor
IGFBP1: Insulin-like Growth Factor Binding Protein 1
IGFs: Insulin-like Growth Factors
IGF-β: Insulin-like Growth Factor beta
IL: Interleukin
IL-1β: Interleukin-1 beta
IL-8 (CXCL8): Interleukin-8/Chemokine (C-X-C motif) ligand 8
ITIM: Immunoreceptor Tyrosine-based Inhibitory Motif
LIF: Leukemia Inhibitory Factor
MAPK: Mitogen-Activated Protein Kinase
MCP-1: Monocyte Chemoattractant Protein 1
MHC: Major Histocompatibility Complex
MHC-II: Major Histocompatibility Complex II
MIF: Macrophage migration inhibitory factor
MMPs: Matrix Metalloproteinases
MRP: Maternal Recognition of Pregnancy
NETs: Neutrophil Extracellular Traps
NF-κB: Nuclear Factor kappa-light-chain-enhancer of activated B cells
NK: Natural Killer (cells)
NKp46, CD16, CD56, CD94: Natural Killer cell markers
NO: Nitric Oxide
OPN: Osteopontin
P4: Progesterone
PBIE: Persistent Breeding-Induced Endometritis
PD-1: Programmed death 1
PD-L1: Programmed death-ligand 1
PGE2: Prostaglandin E2
PGF2α: Prostaglandin F2 alpha
PTGS2/COX-2: Prostaglandin-Endoperoxide Synthase 2/Cyclooxygenase-2
scRNA-seq: Single-cell RNA sequencing
TGF-β: Transforming Growth Factor beta
TGF-β1: Transforming Growth Factor beta 1
Th1: T helper type 1 (proinflammatory T helper cells)
Th2: T helper type 2 (anti-inflammatory T helper cells)
TIGIT: Immunoreceptor Tyrosine-based Inhibitory Motif ITIM domain
TIMP1: Tissue Inhibitor of Metalloproteinases 1
TIMP1: Tissue Inhibitor of Metalloproteinases 1
TIMPs: Tissue Inhibitors of Metalloproteinases
TNF-α: Tumor Necrosis Factor alpha
TRAIL: Factor-related apoptosis-inducing ligand
Treg: Regulatory T cells
TTR: Transthyretin
uNK: Uterine Natural Killer cells
